# Occurrence of Thromboembolism in Paediatric Patients With Inflammatory Bowel Disease: Data From the CEDATA-GPGE Registry

**DOI:** 10.3389/fped.2022.883183

**Published:** 2022-06-03

**Authors:** Jan De Laffolie, Antje Ballauff, Stefan Wirth, Carolin Blueml, Frank Risto Rommel, Martin Claßen, Martin Laaß, Thomas Lang, Almuthe Christina Hauer, the CEDATA-GPGE Study Group

**Affiliations:** ^1^Department of General Pediatrics and Neonatology, University of Giessen, Giessen, Germany; ^2^Kinderklinik, Helios Klinikum Krefeld, Krefeld, Germany; ^3^Kinderklinik, Helios Klinikum Wuppertal, Wuppertal, Germany; ^4^Department of Paediatrics, Philipps-University Marburg, Marburg, Germany; ^5^Klinikum Links der Weser, Bremen, Germany; ^6^Children’s Hospital, Medical Faculty Carl Gustav Carus, Technische Universität Dresden, Dresden, Germany; ^7^Kinderklinik Regensburg, Regensburg, Germany; ^8^Department of Pediatrics and Adolescent Medicine, Medical University of Graz, Graz, Austria

**Keywords:** IBD, paediarics, inflammatory bowel disease, colitis, Crohn’s disease, thromboembolism, children

## Abstract

**Objective:**

Among patients with inflammatory bowel disease (IBD), the risk of thromboembolism (TE) is increased, representing a relevant cause of morbidity and mortality. In contrast to other extraintestinal IBD manifestations, TE receives much less attention because of its low incidence, estimated at merely 0.4–0.9% in hospitalised children with IBD.

**Methods:**

Cases with TE, as documented in the German-Austrian Paediatric IBD registry gesellschaft für pädiatrische gastroenterologie und ernährung – large paediatric patient registry (CEDATA-GPGE), were analyzed retrospectively. For all patients with signs of TE, a questionnaire was filled in by the treating paediatric gastroenterologist.

**Results:**

Over 10 years, 4,153 paediatric patients with IBD (0–18 years) were registered in the registry, and 12 of them identified with TE. Eight patients were diagnosed with ulcerative colitis (UC), three with Crohn’s disease (CD), and one with IBD-unclassified. The median age at IBD diagnosis was 10 years and at the manifestation of TE 13 years, respectively, with a median latency to TE of 2 years. Prevalence of TE was 0.3%, with a significantly higher risk for patients with UC than CD (OR 5.9, CI 1.56–22.33, *p* = 0.008). More girls than boys were affected (f:m = 7:5) without reaching significance. Approximately 90% of patients experienced TE during active disease, with relevant cerebral and limb involvement in 6/12 patients. Various risk factors, e.g., hospitalisation, coagulopathy, or anaemia were identified. TE management included intensive care and surgery. Among the 12 patients, 11 recovered fully, in which one patient has focal epilepsy as a sequela.

**Conclusion:**

Paediatric patients with IBD have a substantially increased risk for TE. Risk factors, such as those identified should be considered when managing paediatric IBD and preventive measures for those hospitalised taken routinely. Initiating pharmacological thromboprophylaxis is challenging for the lack of published trials on efficacy and safety in paediatric IBD but should be considered carefully in each case.

## Introduction

Inflammatory bowel diseases (IBD), consisting of Crohn’s disease (CD), ulcerative colitis (UC), and unclassified IBD (IBD-U), are chronic inflammatory disorders affecting the small and large intestines, with frequent extraintestinal manifestations (EIM) (1–7).

Overall, thromboembolism (TE) is a rare but potentially life-threatening event in children and adolescents. The annual incidence in children in general, excluding neonates, is reported to be 0.05–0.07 per 10,000 children. Paediatric TE includes a heterogeneous population with variable age, localisation of thrombosis, and basic comorbidities. To date, treatment is based on adult trials and expert opinion, due to a lack of evidence in children (8).

The mechanisms by which IBD causes TE are multifactorial and not fully understood (9). However, data suggest that an interplay and dysregulation of various systems lead to TE. IBD is known to involve a complexity of pro-inflammatory mediators that could contribute to a hypercoagulable state (10, 11). Furthermore, coagulation factors, including fibrinogen and fibrin products, are elevated in an acute flare of IBD, and thrombocytosis is detected in active disease states (12). In addition, lower levels of protein S and antithrombin were measured in patients with IBD (13, 14). A sole effect of inflammation seems unlikely, due to the lack of increased risk for TE in coeliac disease or rheumatoid arthritis (1).

The most frequent genetic factors in thrombophilia are prothrombin G20210A polymorphism, Factor V Leiden, and C677T polymorphism of methylenetetrahydrofolate reductase (MTHFR), less often Protein C and S or antithrombin deficiencies. When comparing these traits in adult populations with and without TE, no significant increase in prevalence rate was found (15, 16).

The risk of developing IBD is reduced in patients with inherited bleeding disorders, which could propose a link between vascular thrombosis and intestinal inflammatory processes (17). Although quantitative and qualitative alterations of multiple components of cascade factors, fibrinolytic system, and anticoagulants were discovered, they cannot fully explain the increased TE risk. However, a complex mechanism of amplification of inflammation and coagulation involving platelets and endothelial cells is proposed in patients with IBD (11).

Relevant acquired risk factors include fluid depletion, immobilisation, (emergency) surgery, central venous lines, steroid therapy, oral contraceptives, infectious complications, (cigarette) smoking, and vitamin deficiency (folate, vitamin B6, and B12) with hyperhomocysteinemia (11, 18, 19). A registry-based study on the role of TE in a large European cohort of paediatric patients with IBD gesellschaft für pädiatrische gastroenterologie und ernährung – large paediatric patient registry (CEDATA GPGE) is overdue (20).

This analysis aimed therefore to retrospectively assess TE prevalence in children and adolescents enrolled in the CEDATA GPGE database from Germany and Austria to gain further insights into TE risk in the paediatric population diagnosed with IBD.

### Patients and Methods CEDATA-GPGE Registry

A retrospective analysis of all TE cases as documented in the German-Austrian paediatric IBD registry CEDATA GPGE was performed within the first 10 years of enrolment. CEDATA GPGE is a registry with the aim of improving care for paediatric patients with IBD, started in 2004 by GPGE. Data (initial presentation and follow-up, *ad hoc* at least every 6 months, clinical data, laboratory, diagnostic and therapeutic measures, disease course, complications, and outcomes) is entered prospectively by participating centres (>35 centres in Germany and Austria) *via* a safe online environment. It is not representative of German-Austrian Population since the participating centres are mostly tertiary hospitals or specialized clinics, but also some smaller hospitals and clinical settings.

### Questionnaire and Statistics

For all patients with signs of TE as reported in the registry dataset, a questionnaire (Table 1), including relevant clinical and follow-up data, was completed by the treating paediatric gastroenterologist. Incidence and prevalence were calculated, and disease activity was assessed using standard scores (Pediatric Ulcerative Colitis Activity Index, PUCAI; Pediatric Crohn’s Disease Activity Index, PCDAI). Prevalence was calculated as affected individuals divided by the number of patients in the registry (%). The incidence rate was calculated as the number of patients affected divided by the number of patients and time of observation (per 10,000 person-years). Disease activity scores were retrospectively calculated from prospectively acquired data. Group comparison was performed with the Chi-Square test and the Mann–Whitney U-test where appropriate, Odds ratios (OR) and 95% CIs were calculated.

**TABLE 1:** Items on the questionnaire for treating paediatric gastroenterologists on TE events.

Sex
Diagnosis – Crohn’s Disease/Ulcerative Colitis/IBD-U
Age at first diagnosis of IBD
Age at thromboembolic event
Activity index at the time of TE (PUCAI/PCDAI)
Paris Classification at time of TE
Therapy at the time of TE
Localisation of TE
Additional risk factors (prothrombotic risk factors, infection, cardiorespiratory disease, other systemic diseases, contraceptive drugs, immobilisation, surgery, and iron deficiency)
Therapy of TE
Outcome of TE

Statistical analysis was performed with R version 4.0.3, where appropriate.

## Results

### Epidemiological Data

In CEDATA GPGE 4,153 patients (age 0–18 years) with IBD were registered. A number of 1,204 patients suffered from UC, 2,671 from CD and 278 from IBD-U. In total, we identified 12 patients with TE (Table 2), with 8 patients diagnosed with UC, 3 with CD, and 1 with IBD-U. The estimated prevalence of TE was.3% [95%CI 0.15–0.48%] for IBD in general, for UC.66% [0.28–1.3%], for CD.11% [0.023–0.32%] and for IBD-U.36% [0.09%–1.9%]. The analyzed registration period amounted to 3,112,097 patient days or 8520, 5 patient years, with an incidence rate for TE of 14.1/10,000 person-years [CI 7–25] (29.1 [CI 14–64] for UC and 5.5 [CI 1–16] for CD) (29.1 for UC and 5.5 for CD).

**TABLE 2:** Characteristics of patients with TE in gesellschaft für pädiatrische gastroenterologie und ernährung – large paediatric patient registry (CEDATA GPGE) over 10 years.

Pat	Diagnosis	PUCAI/PCDAI	Paris classification	Medication	Localization of TE	Immo-bilisation	Hemoglobin (g/dl)	Outcome
1	UC	70	E4	No compliance	Venous	Yes	7,5	Rest ad integrum
2	IBD-U	30 (PCDAI)	L2	Aza, Pred, Infliximab	Venous sinus thrombosis	No	7,8	Rest ad integrum
3	CD	5	L3	6-Mercaptopurin	Venous	Yes	11,5	Rest ad integrum
4	CD	53	L2L4a	Aza, Pred	Intracardial right ventricle	Yes	9,9	Rest ad integrum
5	CD	30	L2L4a	Aza	Venous peripheral	No	10,0	Rest ad integrum
6	UC	85	E4S1	Pred, MTX	V basilica (peropheral venous catheter)	Yes	7,0	Rest ad integrum
7	UC	10	E4S0	unknown	Venous peripheral	No	5,1	Rest ad integrum
8	UC	35	E4	Pred, Aza	Vinous sinus thrombosis	No	Unknown	Rest ad integrum
9	UC	Unknown	Unknown	Unknown	Venous peripheral	Unknown	Unknown	Rest ad integrum
10	UC	Unknown	Unknown	Unknown	Venous sinus thrombosis	Unknown	Unknown	Colectomy
11	UC	Unknown	Unknown	Pred, Aza	Myocardial infarction, venous sinus thrombosis	No	Unknown	Reduced left ventricular function, Colectomy
12	UC	5	E4	Pred, Aza, 5-ASA topical	Venous sinus thrombosis, V jug int with hemorrhagic stroke	No	7,4	Focal epilepsy

Patients with UC carried a significantly higher risk for TE compared with patients with CD (OR 5.9, CI 1.56–22.33, *p* = 0.008). More girls were affected than boys (7:5), with a higher risk of developing TE in UC (OR 4.14 [CI.83–20.61], although this trend did not reach significance. Due to the small sample size, estimates were not calculated for CD and IBD-U (f/m 1:2 in CD, only one female with IBD-U affected), nor did we perform a subgroup analysis for age.

### Patients and Situation

Patients experiencing TE had been younger at IBD diagnosis compared with most patients registered, with 50% diagnosed before the age of 10 years (range 2–14 years). The median age at TE manifestation was 13 years (range 2.9–17), with a median latency to TE of 2 years (range 0–10 years).

At TE manifestation, median PUCAI in 5/8 patients was 35 (range 5–70) and median PCDAI 30 (5–53). According to the Paris classification, all patients had colonic involvement, five of them with pancolitis (E4), three patients with CD were L2, two had upper GI involvement, and one was classified as L3. No structuring or penetrating behaviour was observed.

At the occurrence of TE, 6/12 patients were under drug therapy with prednisolone because of IBD activity. Among whom, six patients received azathioprine, one mercaptopurine, one patient with CD methotrexate, and another patient with CD Infliximab. One patient was on no relevant therapy due to non-adherence, whereas in two patients, data on treatment at TE manifestation were inconclusive.

Furthermore, five patients had sinus venous thrombosis, in one patient thrombosis involved peripheral veins, one patient developed a large right ventricular thrombus, and one suffered from myocardial infarction and left ventricular thrombus. Exactly 4/12 patients had been immobilised (restricted to bed-rest) and one patient had received contraceptive medication and underwent surgery 3 weeks prior to TE, with low molecular weight heparin (LMWH) prophylaxis. The Median Hb level was 8.9 g/dl (8/12 patients; range 5.1–11.5 g/dl). In 9/12 patients a thrombophilia screening had been performed: Protein S deficiency was found in two patients, one of whom had presented with right ventricular thrombus, and resistance to activated protein C (APC) was diagnosed in one patient.

### Therapy and Follow-Up

Data on therapy of TE were obtained in 9/12 patients with treatment, including LMWH (7/8) or heparin (1/8) and cumarine in the patient with APC resistance. There was one patient who received cardiothoracic surgery for the large right ventricular thrombus, while the patient with myocardial infarction received coronary angioplasty. Two patients with UC underwent colectomy after TE.

Recurrence occurred in one girl (8.3%), who developed an extensive left ventricular thrombosis 1 week after myocardial infarction, a thrombophilia screening not having revealed any inherited disease/trait. All patients recovered fully from the event, with the exception of one patient with venous sinus thrombosis who has focal epilepsy as a sequela.

## Discussion

To date, the risk of TE in paediatric IBD has been addressed in three studies only, namely of US, Canadian, and Danish cohorts, respectively, and was found to be much higher than in paediatric patients without IBD (5, 21, 22). One reason for the scarcity of data on this particular complication may be that TE only very rarely occurs in the paediatric population, with an annual incidence of 0.7–1.4 per 100,000 children or adolescents and 5.3/10,000 hospitalisations (23, 24). Based on US in-patient data by means of an aggregated, stratified random sample across five time periods, Nylund et al. (25) reported the following cumulative outcome data: The absolute risk for any TE was low, with 50.4/10,000 hospitalizations in children without IBD compared with 119.8 in CD and 101.7 in UC (RR 2.37[2.16–2.61], 1.99[1.51–2.64]), respectively.

Inflammatory bowel disease *per se* is an acknowledged risk factor with pathogenic and clinical specifics accountable for increased TE events, as shown in adult patients with an Odds Ratio of 1.85 [1.7–2.01] in UC and 1.48 [1.35–11.62] in CD, resulting in excess mortality of 2.1-fold compared with patients without IBD (26). The risk seems to multiply during moderate to severe flares with a hazard ratio of 4–8 (27), wherein patients with IBD in active disease sharing, for example, thrombocytosis as a significant trigger of TE (28), and especially those < 40 years having a higher risk of cardiovascular disease (29).

With regard to IBD sub-entities, a paediatric review revealed UC as significantly more frequently associated with TE than CD (OR 3.7 [1.8–7.6]) (23). This was questioned by Barclay et al. in 2010 (3), who compared reports published before and after 2000 and suggested a secular trend toward TE in CD. Data that are more recent do not show any significant trend toward higher rates for TE in UC when compared with IBD in general (22), which is, however in contrast to our findings.

When comparing our study group with the Danish cohort (21), TE incidences vary considerably by age, e.g., in the Danish study, this rate was 2/10,000 person-years in healthy controls < 20 years, 9.8 in UC, 7.8 in CD, and 8.9 in IBD. The TE incidence in our slightly younger study group (<18 years) was higher, with 14/10,000 person-years (5.5 in CD, 29.1 in UC). A further explanation for this higher rate could be the very nature of CEDATA GPGE, as mainly patients from tertiary or large paediatric IBD centres caring for more severe cases are included in this registry. In the Canadian cohort of 3,593 patients with IBD, TE incidence was 81.16 (1 year) and 31.18 (5 years) per 10,000 person-years. TE incidences were particularly high in UC patients, with a higher risk for deep vein thrombosis and pulmonary embolism than in healthy controls (the latter observation was also true for CD patients). Overall, this cohort’s TE incidences were approximately two and three times higher than those of our and the Danish studies, respectively. In adult populations studied, the relatively younger patients were at higher risk of experiencing TE and had a higher risk of recurrence (30, 31). When comparing age at TE, we did not find any relevant differences between patients affected, but the share of those who had been diagnosed with IBD under the age of 10 years was significantly higher.

In a review from 2011 (23), Lazzerini et al. found that 38% of patients affected were younger than 12 years and 11% younger than 6 years, a proportion which is confirmed by our observations. Regarding latency until TE, the authors reported a median of almost 2 years with a wide range (1 month–8 years), which is again in-keeping with our findings (median 2 years, range 0–10 years). As was shown in our study, data from the Canadian cohort demonstrated a higher incidence of TE in the first year after IBD diagnosis, obviously due to high disease activity and induction therapy (e.g., corticosteroids). While, in some contrast to this observation, among adult patients up to 30% experienced TE during well-controlled disease (27, 32), TE in paediatric IBD was in fact mostly associated with active disease, e.g., in some 82.8% of children described (25). This correlates excellently with our own findings, since all but one patient showed clinically active inflammation at the time of TE (90%), although only one CD patient had a PCDAI > 30 and 2 UC patients had a PUCAI > 45, i.e., the majority of our patients showed moderate disease activity.

Disease extension may also play a role regarding the risk for TE, as was shown in adult and paediatric studies. Not only complications like fistulas or strictures are associated with an increased risk, but especially pancolonic involvement in UC and colonic involvement in CD (26), a notion further substantiated by our findings, since all patients affected had colonic involvement. There was no statistically significant difference in rates between girls and boys (29).

The most common sites for relevant TE are deep venous thrombosis of the lower limb, pulmonary embolism, and superior sagittal sinus thrombosis with an incidence of venous TE of some 2 per 1,000 persons per year in Western populations (33, 34). In a paediatric cohort, 54.3% suffered from cerebral, 26% from limb, 13% from abdominal, and 3.3% from retinal and pulmonary involvement, respectively (23). In our cohort, the distribution was somewhat similar, with half of the patients experiencing cerebral, and limb involvement, as well as extended large intracardial thrombosis, but neither retinal nor pulmonary embolism.

Recurrence is reported in 11% during the same hospitalisation, while 10% suffer later from a second episode. In our cohort, 1/12 patients experienced a second TE 1 week after myocardial infarction, reflecting the percentage indicated in the literature.

Fortunately, in our cohort there was no mortality, so we cannot comment on mortality rates that are reported to be significantly higher in patients with IBD (OR 2.5 [1.83–3.43]) (26).

### Therapeutic Consequences

In 2010, the American Gastroenterological Association proposed screening and prophylaxis for TE as quality indicators. Obviously, any prophylactic or therapeutic option should be based on a patient-specific assessment of risk factors, especially in the paediatric population.

Since hospitalisation carries substantial risk, immobilisation should be as short as possible, and the use of supportive stockings is a rule (35). Although there is no evidence in pIBD cohorts, it is acknowledged as risk-reducing in the general population and does not carry a relevant risk for adverse events or high. Currently, and unlike in the case of adult patients with IBD, there is no consensus on the use of LMWH in the paediatric age group. However, in European practice guidelines, the usage of thromboprophylaxis is recommended in patients with severe UC and one or more risk factors (36). In adult patients, current data suggest that prophylactic LMWH is not widely accepted and is underused in patients with IBD (37). Levartovsky et al. conclude from data of an adult cohort that thromboprophylaxis should be given to all patients with IBD hospitalised (38). Scharrer et al. found the risk for major bleeding, albeit not fatal, increased in patients with IBD receiving thromboprophylaxis (39). In any case, a higher risk for TE and its consequences must be taken into account (39).

In case of symptoms suggestive of TE, particularly with cerebral involvement, emergency (neuro-)imaging should be available, and treatment should adhere to national guidelines, with close cooperation with other specialists, e.g., paediatric neurologists. In selected cases, short to long-term prophylaxis with oral anticoagulants should be considered.

### Limitations

Limitations of this study include the nature of a retrospective questionnaire, while data entry in the CEDATA GPGE registry is prospective. Also, the cohort studied consists of well-documented paediatric patients with IBD, which might lead in so far to an overestimation of TE, because most of the patients registered are from tertiary care centres, caring for more complicated disease courses. The scope and influence of missing values, as the question of representation or conclusions toward a general population, cannot be addressed adequately. Furthermore, the conclusion is limited with only 12 patients diagnosed with thromboembolism (9 patients with a full dataset available), however number of detected patients is corresponding to the published data (5, 21, 22).

## Conclusion

In keeping with previous reports on a rise in both TE and IBD incidences, with a higher risk of morbidity and even mortality in younger patients with IBD (morbidity OR 3.22–4.3/mortality OR 2.1) (3, 31), the findings of our study underline that these patients have a substantially increased risk for TE. Consequently, when managing paediatric IBD, an active search for TE risk factors must be undertaken and preventive measures taken, particularly for those hospitalised. Initiation of pharmacological thromboprophylaxis remains a challenge as there are no published trials for the efficacy and safety of primary pharmacological thromboprophylaxis in paediatric IBD. Further studies are needed to clarify the risks and benefits of thromboprophylaxis in this patient group and to identify the most important risk constellations for developing TE.

## Data Availability Statement

The raw data supporting the conclusions of this article will be made available by the authors, without undue reservation.

## Author Contributions

AB, AH, and JD contributed to the conception and design of the study and the CEDATA-GPGE study group acquired the data. SW, CB, MC, ML, and TL provided the additional insights and information. CB, FR, JD, and AH analyzed and interpreted the data. JD, FR, and AH drafted the article and revised it critically for important intellectual content. All authors contributed to the article and approved the submitted version.

## Conflict of Interest

The authors declare that the research was conducted in the absence of any commercial or financial relationships that could be construed as a potential conflict of interest.

## Publisher’s Note

All claims expressed in this article are solely those of the authors and do not necessarily represent those of their affiliated organizations, or those of the publisher, the editors and the reviewers. Any product that may be evaluated in this article, or claim that may be made by its manufacturer, is not guaranteed or endorsed by the publisher.

## References

[1] Miehsler W Reinisch W Valic E Osterode W Tillinger W Feichtenschlager T Is inflammatory bowel disease an independent and disease specific risk factor for thromboembolism? *Gut.* (2004) 53:542–8. 10.1136/gut.2003.025411 15016749 PMC1773996

[2] Zitomersky NL Verhave M Trenor CC III. Thrombosis and inflammatory bowel disease: a call for improved awareness and prevention. *Inflamm Bowel Dis.* (2011) 17:458–70. 10.1002/ibd.21334 20848518

[3] Barclay AR Keightley JM Horrocks I Garrick V McGrogan P Russell RK. Cerebral thromboembolic events in pediatric patients with inflammatory bowel disease. *Inflamm Bowel Dis.* (2010) 16:677–83. 10.1002/ibd.21113 19824070

[4] Bargen JA Barker NW. Extensive arterial and venous thrombosis complicating chronic ulcerative colitiS. *Arch Intern Med.* (1936) 58:17–31.

[5] Mitchel EB Rosenbaum S Gaeta C Huang J Raffini LJ Baldassano RN Venous thromboembolism in pediatric inflammatory bowel disease: a case-control study. *J Pediatr Gastroenterol Nutr.* (2021) 72:742–7. 10.1097/MPG.0000000000003078 33605670 PMC9066981

[6] Lloyd-Still JD Tomasi L. Neurovascular and thromboembolic complications of inflammatory bowel disease in childhood. *J Pediatr Gastroenterol Nutr.* (1989) 9:461–6. 10.1097/00005176-198911000-00012 2621524

[7] Standridge S de los Reyes E. Inflammatory bowel disease and cerebrovascular arterial and venous thromboembolic events in 4 pediatric patients: a case series and review of the literature. *J Child Neurol.* (2008) 23:59–66. 10.1177/0883073807308706 18184942

[8] Monagle P Chan AKC Goldenberg NA Ichord RN Journeycake JM Nowak-Gottl U Antithrombotic therapy in neonates and children: antithrombotic therapy and prevention of thrombosis, 9th ed: American college of chest physicians evidence-based clinical practice guidelines. *Chest.* (2012) 141(Suppl. 2):e737S–e801S. 10.1378/chest.11-2308 22315277 PMC3278066

[9] Guan QA. Comprehensive review and update on the pathogenesis of inflammatory bowel disease. *J Immunol Res.* (2019) 2019:7247238. 10.1155/2019/7247238 31886308 PMC6914932

[10] Brynskov J Nielsen OH Ahnfelt-Ronne I Bendtzen K. Cytokines (immunoinflammatory hormones) and their natural regulation in inflammatory bowel disease (Crohn‘s disease and ulcerative colitis): a review. *Dig Dis.* (1994) 12:290–304. 10.1159/000171464 7882549

[11] Giannotta M Tapete G Emmi G Silvestri E Milla M. Thrombosis in inflammatory bowel diseases: what‘s the link? *Thromb J.* (2015) 13:14. 10.1186/s12959-015-0044-2 25866483 PMC4393581

[12] Kume K Yamasaki M Tashiro M Yoshikawa I Otsuki M. Activations of coagulation and fibrinolysis secondary to bowel inflammation in patients with ulcerative colitis. *Intern Med.* (2007) 46:1323–9. 10.2169/internalmedicine.46.0237 17827828

[13] Collins CE Cahill MR Newland AC Rampton DS. Platelets circulate in an activated state in inflammatory bowel disease. *Gastroenterology.* (1994) 106:840–5. 10.1016/0016-5085(94)90741-2 8143990

[14] Zezos P Kouklakis G Saibil F. Inflammatory bowel disease and thromboembolism. *World J Gastroenterol.* (2014) 20:13863–78. 25320522 10.3748/wjg.v20.i38.13863 PMC4194568

[15] Vecchi M Sacchi E Saibeni S Meucci G Tagliabue L Duca F Inflammatory bowel diseases are not associated with major hereditary conditions predisposing to thrombosis. *Dig Dis Sci.* (2000) 45:1465–9. 10.1023/a:1005541028045 10961731

[16] Mahmood A Needham J Prosser J Mainwaring J Trebble T Mahy G Prevalence of hyperhomocysteinaemia, activated protein C resistance and prothrombin gene mutation in inflammatory bowel disease. *Eur J Gastroenterol Hepatol.* (2005) 17:739–44. 10.1097/00042737-200507000-00008 15947551

[17] Thompson NP Wakefield AJ Pounder RE. Inherited disorders of coagulation appear to protect against inflammatory bowel disease. *Gastroenterology.* (1995) 108:1011–5. 10.1016/0016-5085(95)90197-3 7698567

[18] Bence CM Traynor MD Jr. Polites SF Ha D Muenks P St Peter SD The incidence of venous thromboembolism in children following colorectal resection for inflammatory bowel disease: a multi-center study. *J Pediatr Surg.* (2020) 55:2387–92. 10.1016/j.jpedsurg.2020.02.020 32145975

[19] McKie K McLoughlin RJ Hirsh MP Cleary MA Aidlen JT. Risk Factors for venous thromboembolism in children and young adults with inflammatory bowel disease. *J Surg Res.* (2019) 243:173–9. 31181463 10.1016/j.jss.2019.04.087

[20] Chien KA Hammad HT Gerber L Sheth S Sockolow R Kucine N. Pediatric gastroenterologists’ approach to venous thromboembolism prophylaxis in pediatric inflammatory bowel disease. *J Pediatr Gastroenterol Nutr.* (2018) 66:286–8. 10.1097/MPG.0000000000001690 28742719 PMC6612280

[21] Kappelman MD Horvath-Puho E Sandler RS Rubin DT Ullman TA Pedersen L Thromboembolic risk among danish children and adults with inflammatory bowel diseases: a population-based nationwide study. *Gut.* (2011) 60:937–43. 10.1136/gut.2010.228585 21339206

[22] Kuenzig ME Bitton A Carroll MW Kaplan GG Otley AR Singh H Inflammatory bowel disease increases the risk of venous thromboembolism in children: a population-based matched cohort study. *J Crohns Colitis.* (2021) 15:2031–40. 10.1093/ecco-jcc/jjab113 34175936 PMC8684458

[23] Lazzerini M Bramuzzo M Maschio M Martelossi S Ventura A. Thromboembolism in pediatric inflammatory bowel disease: systematic review. *Inflamm Bowel Dis.* (2011) 17:2174–83. 10.1002/ibd.21563 21910180

[24] Kuhle S Massicotte P Chan A Adams M Abdolell M de Veber G Systemic thromboembolism in children. data from the 1-800-NO-CLOTS consultation service. *Thromb Haemost.* (2004) 92:722–8. 10.1160/TH04-04-0207 15467901

[25] Nylund CM Goudie A Garza JM Crouch G Denson LA. Venous thrombotic events in hospitalized children and adolescents with inflammatory bowel disease. *J Pediatr Gastroenterol Nutr.* (2013) 56:485–91. 10.1097/MPG.0b013e3182801e43 23232326

[26] Nguyen GC Sam J. Rising prevalence of venous thromboembolism and its impact on mortality among hospitalized inflammatory bowel disease patients. *Am J Gastroenterol.* (2008) 103:2272–80. 10.1111/j.1572-0241.2008.02052.x 18684186

[27] Grainge MJ West J Card TR. Venous thromboembolism during active disease and remission in inflammatory bowel disease: a cohort study. *Lancet.* (2010) 375:657–63. 10.1016/S0140-6736(09)61963-2 20149425

[28] Ollech JE Waizbard A Lubetsky A Kopylov U Goren I Dotan I Venous thromboembolism among patients with inflammatory bowel diseases is not related to increased thrombophilia: a case-control study. *J Clin Gastroenterol.* (2022) 56:e222–6. 10.1097/MCG.0000000000001578 34231498

[29] Zhang H Wang X. Risk factors of venous thromboembolism in inflammatory bowel disease: a systematic review and meta-analysis. *Front Med (Lausanne).* (2021) 8:693927. 10.3389/fmed.2021.693927 34262920 PMC8273255

[30] Grip O Svensson PJ Lindgren S. Inflammatory bowel disease promotes venous thrombosis earlier in life. *Scand J Gastroenterol.* (2000) 35:619–23. 10.1080/003655200750023589 10912662

[31] Novacek G Weltermann A Sobala A Tilg H Petritsch W Reinisch W Inflammatory bowel disease is a risk factor for recurrent venous thromboembolism. *Gastroenterology.* (2010) 139:779–87,87e1. 10.1053/j.gastro.2010.05.026 20546736

[32] Papa A Tursi A Danese S Rapaccini G Gasbarrini A Papa V. Venous thromboembolism in patients with inflammatory bowel disease: the role of pharmacological therapy and surgery. *J Clin Med.* (2020) 9:2115. 10.3390/jcm9072115 32635542 PMC7408761

[33] Weitz JI Prandoni P Verhamme P. Anticoagulation for patients with venous thromboembolism: when is extended treatment required? *TH Open.* (2020) 4:e446–56. 10.1055/s-0040-1721735 33376944 PMC7758152

[34] Heit JA Spencer FA White RH. The epidemiology of venous thromboembolism. *J Thromb Thrombolysis.* (2016) 41:3–14. 26780736 10.1007/s11239-015-1311-6 PMC4715842

[35] Solem CA Loftus EV Tremaine WJ Sandborn WJ. Venous thromboembolism in inflammatory bowel disease. *Am J Gastroenterol.* (2004) 99:97–101. 14687149 10.1046/j.1572-0241.2003.04026.x

[36] Turner D Ruemmele FM Orlanski-Meyer E Griffiths AM de Carpi JM Bronsky J Management of paediatric ulcerative colitis, part 1: ambulatory care-an evidence-based guideline from european crohn’s and colitis organization and European society of paediatric gastroenterology, hepatology and nutrition. *J Pediatr Gastroenterol Nutr.* (2018) 67:257–91. 30044357 10.1097/MPG.0000000000002035

[37] Papa A Papa V Marzo M Scaldaferri F Sofo L Rapaccini GL Prevention and treatment of venous thromboembolism in patients with IBD: a trail still climbing. *Inflamm Bowel Dis.* (2015) 21:1204–13. 10.1097/MIB.0000000000000310 25581834

[38] Levartovsky A Barash Y Ben-Horin S Ungar B Klang E Soffer S Thromboprophylaxis for hospitalized patients with inflammatory bowel disease-are we there yet? *J Clin Med.* (2020) 9:2753. 10.3390/jcm9092753 32858826 PMC7565590

[39] Scharrer S Primas C Eichinger S Tonko S Kutschera M Koch R Inflammatory bowel disease and risk of major bleeding during anticoagulation for venous thromboembolism. *Inflamm Bowel Dis.* (2021) 27:1773–83. 10.1093/ibd/izaa337 33386735

